# Microvascular Experimentation in the Chick Chorioallantoic Membrane as a Model for Screening Angiogenic Agents including from Gene-Modified Cells

**DOI:** 10.3390/ijms23010452

**Published:** 2021-12-31

**Authors:** Donna C. Kennedy, Barbara Coen, Antony M. Wheatley, Karl J. A. McCullagh

**Affiliations:** Department of Physiology, School of Medicine, Human Biology Building, National University of Ireland, H91 W5P7 Galway, Ireland; d.kennedy12@nuigalway.ie (D.C.K.); barbara.coen@nuigalway.ie (B.C.)

**Keywords:** chorioallantoic membrane (CAM), angiogenesis, blood flow, cancer, tumour, microcirculation

## Abstract

The chick chorioallantoic membrane (CAM) assay model of angiogenesis has been highlighted as a relatively quick, low cost and effective model for the study of pro-angiogenic and anti-angiogenic factors. The chick CAM is a highly vascularised extraembryonic membrane which functions for gas exchange, nutrient exchange and waste removal for the growing chick embryo. It is beneficial as it can function as a treatment screening tool, which bridges the gap between cell based in vitro studies and in vivo animal experimentation. In this review, we explore the benefits and drawbacks of the CAM assay to study microcirculation, by the investigation of each distinct stage of the CAM assay procedure, including cultivation techniques, treatment applications and methods of determining an angiogenic response using this assay. We detail the angiogenic effect of treatments, including drugs, metabolites, genes and cells used in conjunction with the CAM assay, while also highlighting the testing of genetically modified cells. We also present a detailed exploration of the advantages and limitations of different CAM analysis techniques, including visual assessment, histological and molecular analysis along with vascular casting methods and live blood flow observations.

## 1. Introduction

The appropriate delivery of metabolites and removal of waste products is essential in maintaining tissue homeostasis in the body. For this to occur, the presence of a vast well-connected microvascular blood vessel network is crucial. In the absence of this system, negative effects such as oxygen deprivation and tissue death can occur [1,2]. The microvasculature is also essential for an array of physiological responses including hormone responses and inter-organ communication, and injury responses including immune and inflammatory responses. Angiogenesis is the process by which new blood vessels form from pre-existing vessels, a phenomenon required in normal physiology, development, growth injury and disease [3]. There are two types of angiogenesis, sprouting and intussusceptive angiogenesis. Sprouting angiogenesis is where blood vessels form as a result of sprouts of endothelial cells [4,5]. Intussusceptive angiogenesis was more recently discovered and entails pre-existing blood vessels “splitting” down the middle to form two new branching blood vessels [6]. Angiogenesis is an organised cascade of events, regulated by several pro- and anti-angiogenic growth factors. Pro-angiogenic growth factors include fibroblast growth factor (FGF) [7], vascular endothelial growth factor (VEGF) [8], transforming growth factor-α (TGF-α) [9], TGF-β [10], hepatocyte growth factor [11], and tumour necrosis factor-α (TNFα) [12]. However, thrombospondins (TSP) [13], angiostatin [14] and endostatin [15] can lead to anti-angiogenic effects. The growth of new blood vessels is induced by the delicate balance between pro-angiogenic and anti-angiogenic factors [16]. The release of these factors activates proteolytic enzymes to remodel the extracellular matrix (ECM) of blood vessels, leading to sprouting and reorganisation of new blood vessels [17].

Cancer therapeutic research and the targeting of VEGF have been major impetuses in expanding interest in angiogenesis in more recent times. Nonetheless, our understanding of these angiogenic factors and signalling pathways are still being investigated and more studies are required to fully explore the understanding of the basic mechanisms, and subsequent translation of this to potential therapeutic use. As a result, the development and standardisation of angiogenic assays, both in vitro and in vivo are vitally important in facilitating this research.

Several angiogenic assays have shown particular benefit in the study of microvascularisation, both in vitro and in vivo. These include in vitro assays such as the use of endothelial cells in culture (migration, proliferation, survival and morphogenesis assays), the rat and mouse aortic ring assays, the embryoid body assay and the mouse metatarsal assay [18,19,20,21]. Angiogenic in vivo assays include the corneal micro pocket, the rodent mesentery assay, the dorsal skin fold procedure and the use of subcutaneous sponge/matrix plugs in conjunction with rodent models and the chick chorioallantoic membrane (CAM) assay [22,23,24,25].

**Figure 1:**
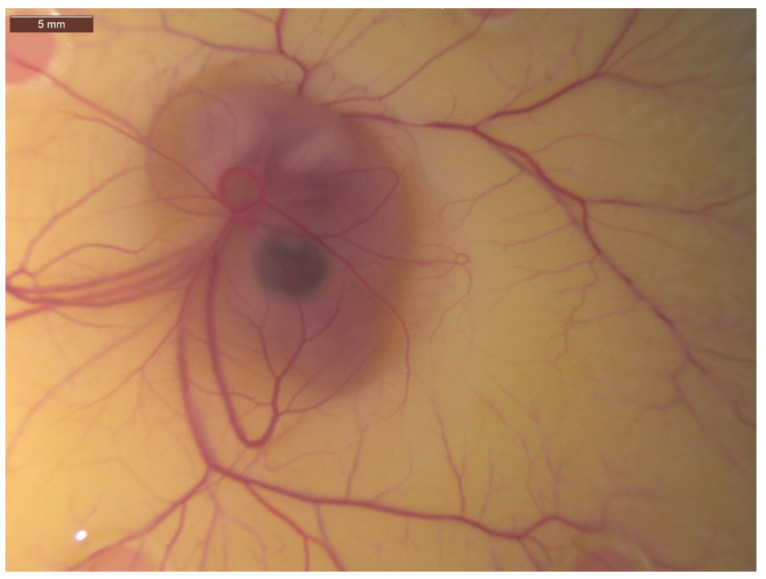
Image of 7-day-old chick embryo with associated chick chorioallantoic membrane (CAM) and its vast vascular network of capillaries, veins and arteries visible. Image taken at 25× magnification.

In vivo investigation is often considered to be more informative than in vitro as it accounts for the interaction of several physiological pathways that cannot be mimicked using cell culture. However, issues such as high-cost, ethical approval and animal sacrifice are drawbacks for most in vivo assays. The CAM assay is an underutilised in vivo angiogenic assay, as it is not subject to these aforementioned drawbacks [26,27]. The CAM is a highly vascularised membrane found in fertilized chicken eggs, with a vast vascular network of capillaries, veins and arteries, which can be easily manipulated and observed for experimental study of angiogenesis (Figure 1) [28,29,30]. The CAM assay can be seen as a bridge which links cell based in vitro studies with in vivo animal experimentation, providing a method to study complex biological procedures while adhering to the “Three R strategy” established by Russell and Burch to reduce animal suffering [31].

## 2. CAM and Chick Development

The CAM is a highly vascularised membrane used for nourishment, gaseous exchange and excretion found in the fertilized eggs of amniotes such as birds and reptiles, analogous to the placenta in mammals [26,30]. The CAM consists of three layers, chorionic epithelium, the mesenchyme epithelium and the allantoic epithelium, each of which carry out their own specific function [30]. The allantoic membrane, derived from the mesoderm is where the primitive blood vessels and vascularisation develop from day 3, with the fusion of the chorionic epithelium and allantoic epithelium occurring at day 4 to produce the double layered chorioallantoic membrane [32]. The CAM consists of several ECM proteins such as laminin, collagen type IV and fibronectin, which allow for the mimicking of the normal physiological microenvironment of warm blooded animals, including humans [33].

Hamburger and Hamilton in 1951 characterised the development of the growing chick embryo, carried out by dividing the 21 days of chick development into forty-six distinctive stages [34]. The CAM grows for the latter 15–16 days of development, expanding alongside the chick embryo until day 21 when the embryo cracks the shell and the egg hatches [30]. Until approximately day 12 the growth of the chick embryo and the CAM vascularisation is undergoing accelerated development. Therefore, the efficacy of any pro-angiogenic or anti-angiogenic factor applied up until this time will be heightened [17]. Consequently, it is recommended to carry out angiogenic assays in the days following day 11, where any new blood vessel generation is more likely resulting from the treatment and not the naturally growing chorioallantoic membrane [27,35].

In the absence of a fully developed immune system until development day 18, the CAM is capable of hosting allogeneic or immune-incompetent acellular matrix or tissue graft until this point. Therefore, the CAM is best employed within a limited window of time in order to accurately assess an angiogenic response and avoid immune reactions. [17,30,36].

**Figure 2:**
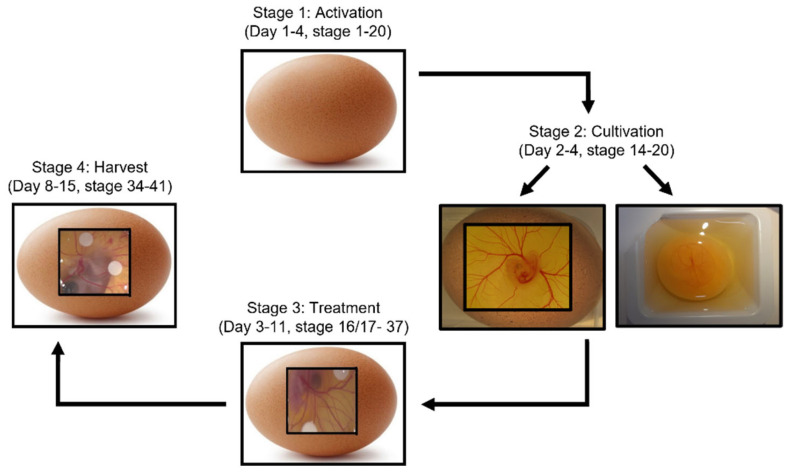
Schematic of four-stage CAM assay process, along with approximate embryonic development days where this stage typically takes place. Stage 1: Activation is where eggs are put in a rotating incubator at 50% humidity to allow for preliminary development. Stage 2: Cultivation allows for visualisation of the embryo and CAM through either ex ovo cultivation where the eggshell is cracked with contents then transferred into a sterile petri dish, or in ovo cultivation where a saw tool is used to excise a window in the surface of the eggshell. Stage 3: Treatments such as cells, drugs or growth factors are applied. This can be through a variety of methods such as application of on-plants, pipetting directly onto the CAM surface or injection into the CAM vasculature. Finally, upon completion of the experiment, the chick embryo is sacrificed and the CAM is removed for analysis. Analysis can include visual observations of angiogenesis, histological examination, or molecular investigation.

The understanding of chick and CAM development is essential for its application as an experimental model. The CAM angiogenic assay procedure follows a basic four-stage process: activation, cultivation, treatment and harvest (Figure 2).

Makanya et al. used light microscopy, ultrastructural analysis and immunohistochemistry to identify and characterise the three specific phases of the 21 day CAM development; phase I (day 8–13, stage 34–39), phase II (day 13–18, stage 39–44) and phase III (day 18–20, stage 44–45) with most rapid growth seen in phase I, less in phase II and even regression observed in phase III [30]. This study reinforces ideas previously expressed by Baum et al. where VEGF-A expression in the CAM peaked during various times in these phases triggering intussusceptive angiogenesis [8]. The understanding of these phases of development has to be considered in the design of a CAM angiogenic assay, with inconsistency possibly leading to hyperinflated interpretation of results in relation to the angiogenic responses observed.

## 3. CAM Assay Procedures

**Figure 3:**
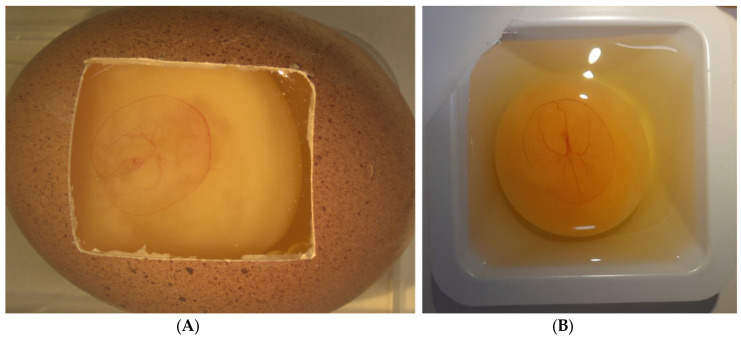
4-day old chick embryo and associated chorioallantoic membrane (CAM) following (**A**) In ovo and (**B**) Ex ovo cultivation. The CAM expands as the embryo grows. Ex ovo cultivation is beneficial through the larger surface area available for experimentation, however embryo survival is impacted.

In the CAM experimental method, there are two basic processes: In ovo cultivation and ex ovo cultivation, based on the Latin for “in the egg” and “outside the egg” respectively (Figure 3). Initially for both methods, eggs are kept in a humidified incubator at a constant humidity and at a temperature of 37 °C for the initial days of development before extraction of the shell from the embryo for visualisation [37].

In ovo cultivation is where a small hole is created at the apex of the egg and 2–3 mL of albumin is removed to lower the embryo and CAM away from the eggshell. A mini-saw tool is used to excise a window in the surface of the eggshell. The created window is covered with a sterile laboratory wrap or plastic cover to ensure sterility and maintain humidity [38,39,40]. The eggs are then returned to a 50–80% humidified incubator for several days before experimentation can begin. This method of cultivation is minimally invasive on the growing chick embryo, providing a relatively unchanged environment for its growth, and generally improves the survival rate for the experiment.

Alternatively, ex-ovo (also referred to as shell-less cultivation) is where the eggshell is cracked or sawed and the embryo, yolk sac and contents of the egg are transferred to a petri dish, cell culture dish or sterile weigh boat and allowed to develop [28,41]. There are few reports of survival rates of embryos during either in ovo or ex ovo cultivation method. However, Dohle et al. have produced a specialised protocol for optimum survival with ex ovo cultivation, indicating survival rates of 50% over 14 days [28]. Lokman et al. report survival with in ovo cultivated embryos of 70% at day 14 [33]. It can be inferred that in ovo shows improved survival compared to shell-less cultivation as it involves less displacement of the embryo, with sterility and humidity issues also reduced. Nonetheless, a limitation with in ovo cultivation is that there is reduced visibility and surface area for experimentation compared to the fully exposed embryo on a petri dish [36].

Naik et al. 2018 suggest an alternative, outlining a detailed protocol where instead of a petri dish or weigh boat, the contents of the eggshell are transferred to a cling film pocket suspended in a plastic cup; this method has shown success, with survivability of >70% reported while still providing the benefit of larger surface accessibility for experimentation [42]. However, despite this apparent success, no other research group has reported use of this method of cultivation to date.

## 4. CAM Experimental Treatments

The CAM has also proven successful with focus on specific areas of research not limited to angiogenic investigation. Successful assays using the CAM have been developed, including but not limited to metastasis, inflammation and propagation, and the grafting of tumour cells [28]. The application of cells in an engraftment onto a CAM was first successfully carried out in 1913, using sarcoma cells to develop tumour growth [43]. Subsequently, the CAM has been subjected to a myriad of treatments, including modified and un-modified cells, tumours, peptides, proteins, plasmids, micro-RNA (miRNA) and pharmacological agents, drugs, metabolites, biomaterials nanoparticles, plant extracts, and growth factors. These treatments were also applied to the CAM by diverse means involving several different scaffolding techniques [44,45].

### 4.1. Scaffolds and Delivery Methods

CAM assay experiments have been adapted to incorporate a wide variety of scaffolding and treatment methods. Outlined in Table 1 are examples of the various scaffolds used in conjunction with the CAM assay, with both biological and non-biological approaches used. Although the test substance in experimentation is important, consideration should also be taken when choosing a suitable scaffold to support the delivery of the treatment. In 2001, Zwadlo-Klarwasser et al. investigated the angiogenic and inflammatory responses of the various biomaterials often used as scaffolds or supports in conjunction with the CAM assay. This research observed increased angiogenesis and cell infiltration due to an inflammatory response when irregular materials such as collagen or filter paper were applied, compared to smoother substances such as PVC or Tecoflex [46]. Considering this, thought should be given when choosing a scaffold, as confounding or skewed results can occur due to the scaffold used or the mechanical influences such as shear stress or stretch, as well as other forces of certain on-plants themselves can trigger an angiogenic response [47,48]. A solution to this issue is the comparison of different scaffolds for the same treatment, as seen in the work by Mangir et al., 2019, where estradiol treatment is applied both by direct pipetting onto the CAM surface and also by encapsulation in a hydrogel scaffold [49]. However, direct application to the surface may not be feasible with certain treatments, such as non-clustering cells, which may require adequate support for survival, and a suitable scaffold to allow containment of cells to within a localised treatment area [50]. The basement membrane matrix Matrigel has been used frequently to fulfil this role. Matrigel, is a basement-membrane matrix taken from Engelbreth–Holm–Swarm mouse sarcomas, consisting of several ECM proteins such as laminin, collagen, heparan sulfate proteoglycans, entactin/nidogen, and a number of growth factors to support cell survival [51,52]. The Matrigel is liquid below 10 °C, therefore it is pre-cooled to mix with cells, and then applied to the warmer CAM surface where it polymerises encapsulating the cells [50]. However, due to the biologically sourced nature of Matrigel, inconsistencies in composition and mechanical properties can occur both between batches and even within batches. This can lead to issues in experimental reproducibility, therefore it has been suggested a move to synthetic polymer scaffold could be a possible resolution [53].

### 4.2. Drugs, Metabolites, miRNAs and Other Treatments

A wide array of both biological and non-biological treatment options has been developed, optimised and applied successfully to the CAM assay. The term biological can apply to both cell and tissue on-plants, or other factors which come from a living source such as hormones, growth factors, metabolites, miRNA and antibodies, with non-biological treatments usually involving drugs, chemicals or nanoparticles.

Outlined in Table 2 are examples of both biological and non-biological substances which have been applied to the CAM assay and have provided a positive angiogenic response, through increased vasculogenesis and neoangiogenesis. In the case of these pro-angiogenic treatments, examples of biological substances are seen much more abundantly, with growth factors such as fibroblast growth factor (FGF), transforming growth factor (TGF) and the known pro-angiogenic vascular endothelial growth factor (VEGF) in several isoforms most commonly described.

Wang et al., investigated the precise mechanism growth factor TGF-β employs to trigger angiogenesis. In this research, the integral role miRNA-29a has in TGF-β induced angiogenesis is explored [130]. MicroRNAs, also known as miRs, are small, non-coding RNA which play a pivotal role in gene regulation [131]. In recent years, it has become evident that miRs play an important role in many cellular processes, including angiogenesis [132]. However, the role miRs have in angiogenic induction or inhibition in the CAM assay has been sparsely investigated. Huan et al., through their research on ameliorating ischaemia in the diabetic foot, have presented and validated a pro-angiogenic miR (miR-21-5p), delivered through exomes [133]. MiR-21-5p induces an increase in vascularisation through upregulation of several angiogenic pathways [133]. Similarly, exomes derived from chronic myeloid leukaemia cells [134] and mesenchymal stem cells [133] also result in stimulating angiogenesis in the CAM.

Direct genetic modification of the CAM is an area also not yet fully explored, with few studies applying gene vectors such as plasmids or recombinant viruses. Transfection of an FGF-1 expression plasmid into the CAM induced significant blood vessel growth [135], and the application of a microgel releasing VEGF-GFP lentivirus vector releasing microgels [136], elicited similar effects, as seen in Table 2.

Although the study of pro-angiogenic treatments focus on the amelioration of vascular conditions such as ischaemia, much of the research involving the CAM assay and anti-angiogenic treatments are oncological, through the reduction in vascularisation of a malignant (growing) tumour [163,164]. Table 3 summarises the application of anti-angiogenic treatments on the CAM assay, which resulted in a range of negative effects in the development of new blood vessels. The success of these anti-angiogenic substances may prove useful in the development of cancer treatments.

Interestingly, anti-angiogenic treatments are frequently seen in literature involve the use of chemical substances, such as the chemotherapy drug doxorubicin [129,165] as well as thalidomide derivatives [59]. The application of nanoparticles such as gold [60,84], green [166], zinc tungstate [61] and chitosan derived nanoparticles [94] have high efficacy in the inhibition of blood vessel development, with significant reductions in blood vessel number, length and density reported.

A method of anti-angiogenic activity with proven potential is the inhibition of various angiogenic growth factors via treatment with targeted antibodies. A WHO approved chemotherapy drug, Avastin^®^ (bevacizumab) is used globally in the treatment of several forms of cancer [167]. Bevacizumaub is an anti-VEGF antibody which has shown potent results by reducing blood vessel density in the CAM assay in several studies [3,80], and other anti-VEGF antibodies have elicited similar effects [168,169]. Equally, the use of anti-human placental growth factor (PGF) [170] and anti-laminin [171] antibodies has led to a significant inhibition of angiogenesis and a delay in blood vessel network development respectively.

The use of the CAM assay for such a variety of both pro-angiogenic and anti-angiogenic factors: drugs, metabolites and biological substances really enforces the efficiency and applicability of the CAM as a screening tool to model the microcirculation and angiogenic effects of various substances.

### 4.3. Cell and Gene Modified Cell On-Plants

In order to ensure successful tumour cell growth, an array of physiological mechanisms such as vessel co-option, intussusceptive microvascular growth, glomeruloid angiogenesis, postnatal vasculogenesis, vasculogenic mimicry and most famously the “angiogenic switch” work together to establish a successful and vast angiogenic network. However, the precise details of many of these mechanisms remain elusive [127,180]. When first applied to the chorioallantoic membrane, tumours undergo a 72 h avascular period before blood vessel infiltration occurs [27].

The use of cell/tumour on-plants in conjunction with the CAM assay has had widespread use. The CAM microenvironment provides all the growth factors, nutrients required for successful cell growth, and the occurrence of the angiogenic switch allows the secretion of Tumour Angiogenic Factors (TAFs) [181] which in turn, induce angiogenesis and allow the penetration of host blood vessels into the applied grafts [182].

Through the injection of cancer cell lines or application of cells with other scaffolds such as Matrigel or collagen encapsulation, or topically through the pipetting or placing of fully formed tumours onto the membrane, the CAM can be used to monitor and investigate the mechanisms of tumour growth, metastasis, and angiogenesis. Based on the current understanding of the angiogenic switch, it is usually expected that following the application of cancer cells or tumour masses onto the CAM, a pro-angiogenic response occurs.

Table 4 outlines examples of un-treated cells, both from cancerous and healthy cell lines, which have been applied to the CAM assay with the aim of observing their angiogenic effect. In the case of most cancer and tumour cell lines, an increase in angiogenic response can be seen, with only some exceptions, such as in the case of SW480 colon carcinoma [151] and Burkitt’s Lymphoma cell lines (BL2) [183] which fail to elicit the anticipated increased vascularisation. Interestingly, the application of non-cancerous cells such as skin grafts and human ovarian tissue can also induce a significant angiogenic response, indicating suitability of the CAM in supporting cell survival.

Angiogenesis is a hallmark of cancer [182], therefore from an oncology aspect, the angiogenic activity of cells is often an area of particular interest, and a potential target area in the development of possible therapeutics or drugs which could hinder this effect. Consequently, the use of the CAM assay as an efficient biological screening tool on the focus of cell induced angiogenesis could prove paramount. Following genetic or other forms of modifications of cells, the changes in cell behaviour or the surrounding media (conditioned media) taken from cells can be an exciting area of focus. Table 5 outlines examples of various cell lines which have been treated or genetically modified to elicit a different angiogenic behaviour compared to their un-modified counterparts.

Once cells are modified to inhibit their pro-angiogenic ability, the CAM assay can be employed as a confirmation tool in order to highlight the efficacy and mechanism of action of the modification. Alternatively, cells can be altered to increase their angiogenic potential, through inhibition or overexpression of certain genes. The efficacy of these cells at inducing a pro-angiogenic response can be measured using the CAM assay [194,196,208].

It must also be noted that in the case of certain cell lines, the pro-angiogenic modification to cells may elicit benefits, especially in areas such as cell therapy, where overexpression of vascular factors could provide a treatment option in therapeutic angiogenesis, to improve the vascularisation of previously ischaemic tissue [135].

Methods of adjusting the angiogenic potential of cells can vary from pre-treating cells with drugs or inflammatory factors such as sphingosine-1-phosphate [193], interferon or tumour necrosis factor [196], to more complex methods of genetically modifying the angiogenic behaviour of a cell.

Studies have used gene expression plasmids to stably transfect cell lines, establishing cells which overexpress various angiogenic or anti-angiogenic factors. The gene modified cells, or the conditioned media of these cells is applied onto the CAM assay respectively.

Examples of angiogenic expression plasmids transfected into cells include FGF plasmid transfection into bovine endothelial cells [135] and argonaute-2 transfected into myeloma cells [195], both of which lead to significant increases in capillary and infiltrating blood vessel numbers following cell-mediated delivery onto the CAM assay. Conversely, the cell-mediated inhibition of blood vessel formation can be seen with transfected anti-angiogenic expression plasmids such as vascular endothelial cell growth inhibitor (VEGI) [203], endostatin [200], or the novel immunotoxin (pVEGF165PE38-IRES2EGFP) [204].

In recent years, gene modifying agents including miRNAs (miRs) have become very popular therapeutic targets. Overexpression or inhibition of various miRs can impact on angiogenic potential of cells through a variety of means. Zhang et al. used an expression plasmid to inhibit the anti-angiogenic function of miR-338 in hepatocellular carcinoma, leading to a significant increase in small blood vessel formation in the CAM assay [194]. Conversely, Li et al. overexpressed miR-181a-5p in fibrosarcoma (HT1080) cells leading to a reduction in CAM blood vessel formation [205].

Interestingly, Jiang et al. focused their research on how the overexpression or inhibition of miR-181a-5p could both attenuate and increase the angiogenic potential of endothelial colony-forming cells (ECFCs) in conjunction with the CAM assay. In this study, lentiviral vector inhibition of miRNA-205 in ECFCs led to an increase in blood vessel density. However overexpression of miRNA-205 resulted in visibly reduced blood vessel formation [192].

Short Hairpin RNAs (shRNAs) are small, manufactured RNA molecules with a sharp hairpin turn used to silence or knockdown gene expression through RNA interference (RNAi) [209]. ShRNAs have been used to inhibit several angiogenic genes and miRNAs in cells applied to the CAM assay, with delivery seen both as direct shRNA delivery, or lentiviral mediated delivery into cells. ShRNA inhibition of potent pro-angiogenic genes is seen throughout the literature, through the knockdown of VEGF [201], connective tissue growth factor [199] and angiopoietin-2 [198] resulting in significant reductions in angiogenic effects in the CAM assay, as outlined in Table 5.

The study of cell application onto the CAM assay monitoring angiogenic response is one with much potential. The applicability and ease of use of the CAM assay is proven by the ease at which grafts can be applied and their survival supported. Use of the CAM assay can provide a screening tool for the inherent angiogenic nature of the cells and the effect of gene/chemical modifications on this inherent ability. However, this is an area which still needs much further exploration.

## 5. CAM Analyses

Considering the success of on-plant treatments using the CAM assay, emphasis has focused on the method of analysis chosen to quantify an angiogenic effect. Assessment of the angiogenesis occurring due to stimuli can be carried out by a variety of different methods. Some studies choose to use arbitrary quantification methods, such as visual or macroscopic evaluation or observation of an angiogenic effect between experimental groups [16,121,147] and define the results simply as positive or negative [210]. While other studies indicate a positive or negative angiogenic effect due to the presence or absence of a “spoke-wheel pattern” of blood vessels approaching an on-plant [55,127]. In the majority of studies such as these, assessments are carried out in a blinded manner in order to prevent bias affecting the results [64,90,104]. The use of adequate positive, negative, and internal controls for comparison using the CAM assay is essential. Generally, neutral phosphate buffered saline (PBS) treated vehicles or scaffolds are used as an internal control, while known angiogenic agonists (such as VEGF) and antagonists (bevacizumab) can be used as a positive and negative controls respectively [80].

Some studies have chosen to compare treated areas of the CAM with non-treated areas [190], others compare the angiogenic effect of an internal control against the treatment; an internal control usually is found in the form of an empty scaffold or a scaffold treated with an angiogenic neutral substance, such as PBS or the solvent used for the delivery of the treatment. Comparison of a treated area to an internal control is preferable and more accurate as it results in less variation between test and control, while also considering the angiogenic response that the vehicle alone can induce.

As individual scoring or assessment methods of CAM treatments can result in conscious or unconscious bias in either direction, a multiprong approach of using different imaging, scoring and assessment techniques is recommended, with the cross-referencing and correlation of results obtained essential to create an overall profile of the angiogenic effects.

### 5.1. Sectioning and Staining Techniques

Following the sacrificing of the chick embryos, the CAM tissue can be fixed using paraformaldehyde or other fixative solutions, excised from the embryo, embedded in paraffin and then undergo sectioning or ultra-sectioning in preparation for histochemical staining for various indicators of angiogenesis such as endothelial and smooth muscle cell markers [211]. Blood proteins such as haemoglobin [212], von Willebrand factor [184,213] or the filament protein desmin [56] have been studied as a measure of blood vessel density and consequently, the vascularisation present [183]. Histological staining of endothelial cells with biotin or fluorescent tagged lectins has also been successfully achieved to visualise vascularisation present in CAM tissue [207,214], while immunohistochemical staining of endothelial cells using anti-CD31 antibodies has also been employed [97,215,216].

**Figure 4:**
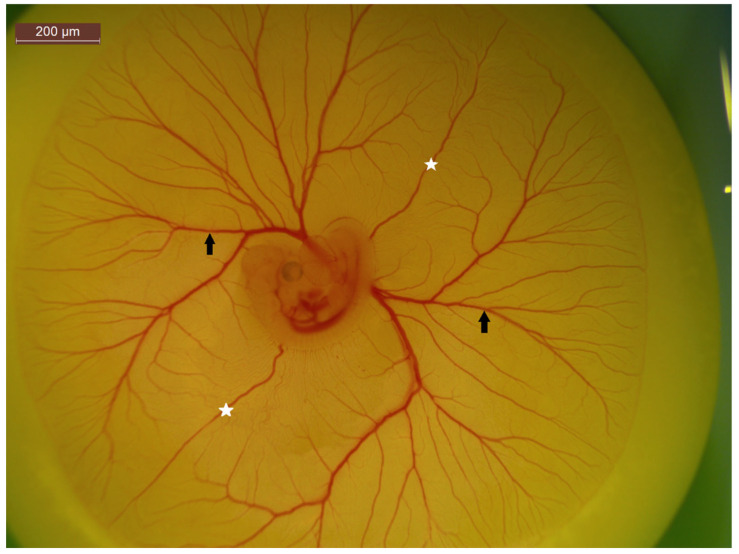
A 5-day-old chick embryo highlight CAM vasculature. The white stars represent anterior and posterior vitelline veins, while the black arrows indicate vitelline arteries and veins. The non-branching nature of the vitelline veins make it an ideal location for injections.

While the quantity of newly formed blood vessels is often the focus of many studies, the quality should also be considered. An issue with some pro-angiogenic factors dependent on dosage levels can be the development of aberrant and leaky blood vessels, inferior to those formed from natural angiogenesis. To investigate this, Pink et al. (2012), developed a modified version of a Miles Assay, a commonly used technique which measures vascular leakage, allowing both the quantity and quality of angiogenesis to be assessed [110,124,217]. In this study, the leakiness of the newly formed blood vessels was quantified by spectrophotometrically measuring the amount of leaked Evan’s blue dye following a single bolus injection. Alternatively, the injection of fluorescent dyes such as various FITC-dextrans of different molecular weights [218,219,220] or FITC and rhodamine conjugated lectins into CAM tissue can prove useful for measurements of vascular leakiness [211]. In this process, fluorescent dyes are injected into the vitelline vein (Figure 4), given time for the dye to circulate, and then observed under fluorescent light, also highlighting smaller capillaries which would otherwise be unquantifiable.

### 5.2. Image Quantification Techniques

Automated, semi-automated and manual serological methods can be used to quantify neovascularisation and angiogenesis. For automated methods, many software packages exist which can be adapted to identify tubules, vessel branch points and network junctions. Such software packages include: Angiotool [221], AngioQuant [60,73,137], Wimasis [87,101], HetCAM [222], Photoshop CS4 [223] and Synedra view [224]. Image analysis software such as Image J can be used for both manual and semi-automated quantification methods. In a semi-automated manner, pixel intensity, or percentage of binary images containing blood vessels can be used to measure blood vessel density [141,225,226]. While manually counting tools can be used to quantify the number of blood vessels, junctions or branching points visible within a defined area [80,227,228].

**Figure 5:**
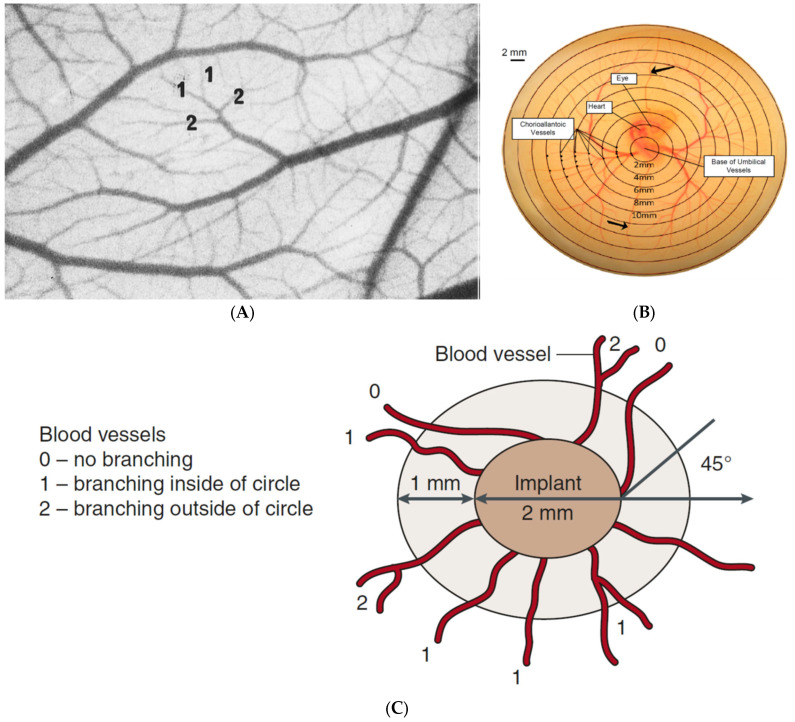
Examples of CAM analysis techniques to quantify angiogenic score following treatment. In the case of each of these methods, each blood vessel which fits specific criteria is given a score, with the accumulative score then determined for each on-plant/treatment. (**A**) Centripetal ordering method of angiogenic scoring, where vessels are assigned a score based on the order of their branching, with higher order vessels getting a higher score as described in DeFouw et al. [231]. (**B**) A range of concentric circles projected onto an image of a CAM where the total vascular index quantified based on the intersection of blood vessels with each of the circle, as described in Burggren et al. [230]. (**C**) Evaluation of a angiogenic response by scoring vessel branching as described by Ribatti et al. [32], this method involves the assigning of an angiogenic score ranging from 0–2 based on branching and angle of approach.

Various parameters can be chosen to assess an angiogenic effect; basic approaches can involve simply counting the blood vessels or quantifying blood vessel length within a circle or square area around an on-plant [215] or converging towards it. More complex methods can involve the scoring of blood vessels based on a centripetal ordering method (Figure 5A), where a blood vessel is order-1, continuous with the capillary network, or order-2, formed from the convergence of two order-1 vessels [178,229]. An alternative method is where an array of concentric circles is projected onto a CAM image with a vascular score then assigned based on the intersection of these circles with blood vessels, without discrimination between arterial or venous vessels (Figure 5B) [37,64,176,177,230].

Ribatti et al., 2007 describes a method where an angiogenic score is assigned to a blood vessel entering an on-plant at a specific angle (Figure 5C) [32,37]. Although, this is a widely used scoring method, issues can arise. Without discrimination of blood flow direction in vessels, determination whether a blood vessel is growing towards or away from an on-plant cannot be fully discerned. Another consideration is that due to the vague nature of branching, scoring and angles as described in this method, individual interpretations and differences in scores can result between analysts studying the same images, leading to erroneous experimental outcomes.

Many image techniques and scoring systems fail to fully explore the changes in microcirculation in response to a treatment. In many studies, the macroscopic observations of larger blood vessels are the main focus, with little attention drawn towards the minute microvessels and capillaries present. This seems to be a major oversight when the study and investigation of pro angiogenic or anti-angiogenic treatments are considered, where even the smallest modifications to vascularisation should be scrutinized.

### 5.3. Vascular Casting

A negative aspect of image quantification and study is that only sporadic random areas of microvascularisation in the CAM are usually observed. However, the development of a three-dimensional microvascular corrosion casting (vascular casting) method could provide an overall thorough study of the vascular changes occurring in the CAM [232]. Corrosion casting is an anatomical method where a solid faithful replica of a biological sample is produced from a hollow anatomical structure or space. In this process, following perfusion to flush out the area, a flexible substance (such as rubber, resin or polyurethane) in liquid form is injected into the space, allowed to solidify and then the surrounding tissue is removed by enzymatic or chemical degradation [233].

This method is particularly useful in the study of vasculature in conjunction with angiogenic assays, where vascularisation and blood vessels distribution and organisation can be measured [234]. Following the production of a vascular cast, examination of the normal or abnormal blood vessel network can be carried out by scanning electron microscopy (SEM), micro computed tomographic (μCT) imaging, or synchrotron radiation-based micro computed tomographic (SRμCT) imaging [235].

In the case of the CAM assay, the chorioallantoic membrane is carefully incised at the central vein, flushed with a sodium chloride (NaCl) and heparin solution to clear the blood, and then perfused with a cast material such as Polyurethane, Clear Flex 95 or Mercox^®^ [236]. Following this, the cast substance is given time to polymerise and then the CAM tissue is dissolved over several days or weeks in 5–20% potassium hydroxide (KOH) or 20% sodium hydroxide (NaOH) followed by rinsing in distilled water or formic acid [234]. Following cast formation, a sputter-coating of gold, silver, platinum, or chromium from 10 nm to 25 nm in thickness may also be required in conjunction with scanning electron microscopy in order to improve image quality [48,235,237].

This method of CAM examination has proven advantageous as it allows for a three-dimensional representation of the vascular network, allowing visualisation of branch points, and indicators of sprouting and intussusceptive angiogenesis [8,48,146], often also being used as a validation method when investigating the efficacy of different imaging techniques [238,239]. As a detailed representation of the CAM vasculature is produced, with the use of the correct casting material, orientation, distribution and frequency of endothelial cells and aberrations in blood vessels can be resolved [234].

### 5.4. Live Blood-Flow Observation

Many of the approaches mentioned previously involve the use of non-viable excised and fixed dead tissue, with observation of morphological characteristics. Several approaches have been developed for the observation of real-time in vivo blood flow and circulation in the CAM. The observation of microcirculation in real-time is advantageous as it can monitor for vascular leaks, variations in vessel quality and density, while also indicating the delivery and efficacy of a treatment. Real-time blood flow can be observed by a wide array of means, including the use of nanoparticles or fluorescently labelled erythrocytes injected into the CAM, or the use of fluorescent dyes or dextrans which are then viewed using intra-vital fluorescent microscopy (IVFM) [29] (Video S1), or alternatively the use of photodynamic therapy (PDT) [85,191].

### 5.5. Molecular Analysis

Although it is useful to observe a visual effect following treatment, molecular analysis of the CAM assay can also prove useful in the understanding of the biochemical mechanisms behind the changes in vascularisation which are observed. To this end, molecular approaches such as quantitative PCR, in situ hybridisation (mRNA), whole mount immunostaining and immunoblotting (protein) can be employed [39].

Quantitative PCR is an essential form of molecular analysis which can prove useful in measurement of changes in gene expression in CAM tissue following various treatments, cells or tumour applications. In some cases, measurement of precise areas around the treatment location are required, therefore laser dissection of CAM tissue can be utilised [240]. Trizol or mRNA isolation kits are often used to extract total RNA from dissected CAM tissue, with qPCR or semi-quantitative PCR then carried out to measure expression of various genes, including those related to angiogenic pathways. In the case of semi-quantitative PCR, following gene amplification, the PCR products are electrophoresed on a polyacrylamide or agarose gel with the band intensities then measured [8,71,214,241]. The use of a suitable chicken specific primer for housekeeping gene expression is paramount in qPCR to ensure accurate, quantifiable results to compare with genes of interest. It also can be used to measure the quality and integrity of the RNA isolated, with β-actin and GAPDH most commonly used in the case of CAM tissue [60,71,134].

In the use of the CAM assay as a model of tumour growth and metastasis, the quantity of human cells present within tissues extracted from chick embryos can be determined by qPCR amplification of the Alu repeat sequences repeats (Alu-qPCR) [54,242]. Alu elements are non-autonomous retrotransposons, which are uniquely present in a primate genome and absent in chicken DNA. Alu PCR can be used a DNA fingerprinting technique to calculate the quantity of human DNA present in CAM tissue [243].

This method has been used in several studies to examine the engraftment and migration of cancer cells from a tumour placed on the CAM surface through the CAM and even into the chick embryo itself, travelling via the vast vascular network present. In summary, to quantify human tumour cell intravasation into the chick CAM, semi-quantitative real time PCR is carried out to amplify Alu sequences in order to calculate the amount of human DNA present in each CAM sample. A standard curve generated by serial dilution of human tumour cells is then used to quantify the actual number of tumour cells present in each CAM sample [17,244,245]. In addition to this analysis, quantification of chick DNA present should be carried out through amplification of a chick house-keeping gene, such as the chick GAPDH genomic DNA sequence [246]. Horst et al. optimised this process, establishing a TaqMan^®^ based quantification method to measure human Alu sequence amplification in genomic DNA from CAM tissue showing improved success compared to the SYBR^®^ Green methods used previously [247].

While study of gene expression can provide insights into the changes occurring in CAM tissue, several studies have instead chosen to quantify the protein expression present by means of Western blotting (immunoblotting). In this process, CAM tissue is crushed or minced, lysed with a suitable lysis buffer such as radioimmunoprecipitation assay (RIPA) buffer. Then the samples are denatured in a suitable loading buffer, such as Laemmli buffer and electrophoresed on an SDS Polyacrylamide gel (SDS-PAGE). Following this, lysates are then transferred to a membrane and probed using various primary antibodies [39,248]. In immunoblotting, use of a suitable protein loading control is imperative, chicken specific α-tubulin and β-actin antibodies have been used in several CAM tissue immunoblots [71,151,249].

Purification of protein extracted from CAM tissue prior to denaturing and SDS-PAGE may be necessary to ensure success. After protein isolation from CAM tissue, immunoprecipitation of samples can be carried out by various means to enrich for the specific protein of interest. Ribatti et al. used Heparin-Sepharose columns to purify protein extracted from CAM tissue prior to immunoblotting and probing for bFGF [250]. Similarly, protein A–Sepharose beads bound with suitable antibodies have also been used with much success [58,251].

Some studies use a two-pronged approach for molecular analysis, using both methods to measure gene, and protein expression. Mangieri et al. used RT-PCR, Western blotting and a visual scoring method to assess increased angiogenesis in the CAM following multiple myeloma endothelial cell treatments. In this investigation, the mRNA expression level of various angiogenic genes, including endostatin, in CAM tissue was measured by qPCR, while Western blotting examined for the altered protein expression of endostatin [249]. Using both methods allowed for correlation of results obtained, where both reduced gene expression and protein secretion of endostatin was observed.

Molecular analysis used in conjunction with other methods of analysis can be extremely useful in investigating the overall effects certain treatments or cell applications can have on the growth and development of the CAM membrane. Immunoblotting can investigate protein secretions, while qPCR examines gene up-regulation or down-regulation. Alternative methods of molecular analysis can include transcriptome analysis of cRNA isolated from CAM tissue following treatments [97]. Gelatin Zymography is another molecular process, where samples are electrophoresed on a polyacrylamide gel containing gelatin, then incubated in collagenase buffer with gelatinolytic activity and finally visualised using 0.5% Coomassie blue [252]. The aforementioned methods are useful in identifying the signalling pathways which result from the application of pro-angiogenic or anti-angiogenic factors.

## 6. Advantages and Limitations of the CAM to Study Microcirculation

Generally, it can be seen that the CAM assay offers many advantages for quantifying angiogenesis over other in vitro and other in vivo methods. Predominantly, the low cost, accessibility, rapid growth and enclosed mechanism of survival make it a clear choice to be used as a research tool [28,33]. The CAM assay is flexible, with a wide variety of treatment methods and delivery options available, with the resulting changes in vasculature assessed by a variety of means. The outputs of a CAM assay can be seen in real time, with the general growth period of the angiogenic window restricted to around developmental day 12. As with all animal models, the CAM has some limitations. Many molecular assessment methods require the acquisition of less widely available chick-specific reagents, antibodies, and probes, as well as specialised equipment and incubators for the experimental process. The developing chick does not have a functioning immune system until development day 18. Therefore, the application of test treatments does not illicit an immune response. However, this can also lead to negative repercussions, the absence of an immune response in the chick can be advantageous, however this lack of protection can hinder the survivability of the chick following invasive techniques such as cultivation (in ovo versus ex ovo). This along with the individual differences between eggs, leads to the requirement of larger sample sizes (n number) of chicks in order to obtain statistical power.

In CAM image analysis, there is large flexibility in the variety of methods for assessment of angiogenic responses. However, this can be misleading as there can be a lack of clarity between different studies as to what determines a significant effect of change in vascularisation. Flexible forms of assessment and arbitrary quantification may not consider natural morphological changes due to the growing embryo alone rather than the treatment applied.

## 7. Concluding Remarks

In the scientific study of microcirculation and angiogenesis, an in vivo approach is very advantageous over in vitro methods. The CAM assay has proven itself to be an invaluable tool in this regard. Presently, the CAM assay is used as a research method in the fields of biology, bioengineering, and chemistry for a wide variety of applications. Although individual laboratory methods, treatment techniques and assessment methods hinder the standardisation of this assay, it does have great potential to be used as an invaluable preliminary and/or complimentary screening tool before examination in higher order animals or a more specific in vivo experimental approach. Overall, the CAM assay is an excellent tool for research, with a low cost, high flexibility, accessibility, with a clear experimental approach. It should be considered greatly for vascular studies before any experimentation using rodents or larger level pre-clinical animal models are commenced. In this regard, the CAM is very much supporting a reduced reliance on pure animal research and following the 3Rs (replacement, reduction, refinement) approach to animal research.

## Figures and Tables

**Table 1:** Examples of various forms of scaffolds and delivery techniques for a variety of pro angiogenic and anti-angiogenic treatments used on the CAM assay.

Scaffold/Delivery Method	Reference
Collagen	[54,55,56,57]
Filter disc	[58,59,60,61,62,63,64,65]
Gelatin sponge	[66,67,68,69,70,71]
Glass discs	[72,73]
Hydrogel	[41,74,75,76,77]
Injected	[78,79,80,81,82,83,84]
Matrigel	[16,85,86,87,88,89,90]
Methylcellulose disc	[15,91,92,93,94]
Microspheres	[95,96]
Pipetted onto surface	[97,98,99,100,101,102,103]
Plastic ring	[75,104,105,106,107,108,109,110,111]
Scaffold	[108,112,113,114,115,116,117]
Thermanox coverslip	[118,119,120,121,122]
Tumour	[5,123,124,125,126]
Pellet	[127,128,129]

**Table 2:** Examples of non-cellular treatments applied to the CAM which elicited a pro-angiogenic response.

Treatment	Delivery Method	Angiogenic Outcome	Ref.
Connective tissue growth factor (CTGF)	Scaffold	Significant increase in blood vessel number and diameter following software quantification	[137]
	Thermanox Coverslips	A dose dependent increase seen by appearance of spoke wheel pattern of blood vessels radiating from on-plants	[138]
Platelet-derived growth factor (PDGF)	Thermanox Coverslips	Macroscopic observations indicated thickening of CAM, but no vascular response	[139]
	Scaffold	An increased blood vessel density converging towards on-plant observed along with thickening of CAM membrane	[140]
Basic Fibroblast Growth Factor (bFGF/FGF-2)	Scaffold		
	Plastic ring	Significant increase in number of blood vessels converging towards on-plant	[110]
	Filter disc	Significant increase in mean fluorescent vascular density, measured by pixel intensity	[141]
		Increased number of branch points in a region around on-plants	[142]
Transforming growth factor-β (TGF-β)	Filter disc	Radial formation of new vessels seen in area around on-plants	[143]
TNFα	Filter disc	Significant increase in tube length and size as measured by angiogenic software	[144]
VEGF-165	Filter disc	A dose responsive increase in blood vessels in defined area observed	[145]
	Hydrogel	A time-dependent increase in blood vessel diameter and branching points, measured using angiogenic software	[146]
	Thermanox Coverslips	Macroscopic observations saw a dose dependent increase in angiogenesis	[139]
VEGF-121	Thermanox Coverslips	Macroscopic observations noticed a change in vascular pattern under the treatment area	[147]
	Filter disc	Software quantified a dose responsive increase in total blood vessel network length	[148]
VEGF-A	Filter disc	Significant increase in sprouting blood vessels within a defined area	[149]
	Scaffold	Increased blood vessel density observed within a defined area	[140]
	Matrigel	Microvascular mapping of the blood vessel network following FITC injection resulted in increased blood vessel density	[150]
	Glass fibre filter disc	Significant increase in vessels number quantified in a random square areas of CAM surface	[151]
	Plastic ring	Angiogenic software indicated a significant increase in number of branchpoints and average vessel length	[111]
	Hydrogel	Significant increase in vessel length, number and vasculogenic index	[74]
		Significant increase in vessel number in a region around on-plant	[152]
VEGF-C	Hydrogel	Significant increase in vessel number in a region around on-plant	[152]
	Methylcellulose disc	Increase in sprouting blood vessels present within a defined area	[153]
Thyroxine	Hydrogel	Significant increase in vascular penetration of on-plants	[154]
Heparin	Hydrogel	Significant increase in vessel number in a region around on-plant	[40]
VEGF-D	Hydrogel	Significant increase in sprouting blood vessels present within a defined area	[152]
Estradiol	Plastic ring	Calculation of percentage of CAM surface covered by endothelial cells resulted in a significant increase in the mean vessels count	[49]
		Angiogenic software quantified a significant increase in number of branchpoints and average vessel length	[111]
	Scaffold	Increase in angiogenic response seen by measurement of vasculogenic index	[117]
Estradiol	Filter disc	Increased vascular branching observed within a defined area	[155]
L-Arginine	Filter disc	The number of primary, secondary, tertiary, and quaternary blood vessels counted with a significant increase in number of quaternary blood vessels	[3]
TGF-β induced miR-29a upregulation	Pipetted	Significant increase in number of blood vessels around on-plant observed	[130]
Fibroblast growth factor-1 expression plasmid	Pipetted	Significant increase in number of blood vessels in a region around on-plant observed	[135]
VEGF-GFP LV	Microgels	Increased blood vessel development quantified	[136]
MSCs-exomes	Not mentioned	Significant promotion of new blood vessel formation	[133]
miR-21-5p OE exomes			
Transthyretin	Plastic ring	Significant increase in number of blood vessels growing towards on-plants	[110]
Terbutaline	Plastic disc	Counting blood vessels which intersected a concentric circle projected around on-plants observed a significant increase in number of blood vessels	[64]
β2AR antagonist	Coverslip	Increased number of blood vessel branch points observed within on-plants	[156]
Angiogenin	Thermonox discs	Visibly increased number of blood vessel spoke wheel pattern seen radiating from on-plants	[157]
Adenosine	Elvax Polymer pellet	Dose dependent increase in vascular density observed in a region around on-plant	[158]
		Observation of spoke wheel pattern of blood vessels radiating from on-plants, with a positive result observed in majority of samples	[127]
ADP			
ATP			
Lactic Acid			
Malate			
Exosomes derived from chronic myeloid leukaemia cells (K562)	Plastic ring	Treatment with a higher concentration resulted in an increase in neovasculature	[134]
2-deoxy-D-ribose	Plastic ring	Angiogenic software calculated a significant increase in number of branchpoints and average vessel length	[111]
Sclerostin	Gelatin sponge	Increased number of blood vessels converging towards on-plants observed	[159]
Roxarsone	Gelatin sponge	Increased number of neovessels and blood vessel length	[160]
Leptin	Gelatin sponge	Software measured significantly increased blood vessel tube length and size	[71]
Arsenic	Filter disc	Dose dependent increase in blood vessel number observed, however higher doses resulted in negative effects	[161]
Y2O3 nanoparticles	Scaffold	Improved blood vessel formation, vascular branching and blood vessel diameter within the area around scaffolds	[162]

**Table 3:** Examples of protein, viral, micro-RNA and pharmacological treatments applied to the CAM which elicited an anti-angiogenic response.

Treatment	Delivery Method	Angiogenic Outcome	Ref.
Nicotinamide adenine dinucleotide (NAD)	10% EVA copolymer Pellet	No spoke wheel pattern was observed radiating from on-plants	[127]
Pyruvate			
Succinate Fumarate citrate			
Avastin (Bevacizumab)	Injected	Significantly less vascular nodes and branches were quantified within a defined area	[80]
EG-VEGF Antibodies		No significant differences in vessel density observed, but dilated medium and large vessels observed	[168]
Methyl blue	Microspheres	No spoke wheel pattern was observed radiating from on-plants	[96]
Chloroquine & Doxorubicin	Agarose pellet	Combination of doxorubicin and chloroquine resulted in strong anti-angiogenic effect on capillaries near on-plants	[129]
Avastin (Bevacizumab)	Pipetted	Significant decrease in percentage of surface area occupied by microvessels	[165]
Vitamin C	Pipetted	The number of primary, secondary, tertiary, and quaternary blood vessels was counted, with decrease in quaternary blood vessels quantified	[3]
MART-10 (Vitamin D analog)	Pipetted	Reduced vessel branch point numbers observed within a defined area	[172]
Green nanoparticles	Gelatin sponge	Decrease in vessels length and branch number within a defined area	[166]
Rhaponticin	Filter disc	Software determined a significant reduction in total blood vessel length	[173]
Thalidomide derivatives	Filter disc	Reduction in vessel number, branch points, neovascularization and total length of vessels	[59]
High affinity PGF-specific Nanobody	Filter disc	Significant inhibition of angiogenesis within a defined area	[170]
Antithrombin	Filter disc	Potent antiangiogenic activity in blood vessel tubules, networks and branching points	[174]
Zinc tungstate nanoparticles	Filter disc	A dose dependent reduction in percentage of surface area occupied by blood vessels was calculated	[61]
Gold nanoparticles	Filter disc	Software determined a dose dependent reduction in blood vessel size, length and branch points	[60]
	Injected	Software determined a significant reduction in vessel length and number of junctions and complexes	[84]
miR-7 mimics	Nitrocellulose rings	A reduction in vascular density within a defined area was visible	[175]
Sunitinib (receptor tyrosine kinase inhibitor)	Nitrocellulose rings	A reduction in vascular density within a defined area was visible	[175]
Vasohibin Adenovirus	Matrigel	Macroscopic observations saw inhibition of blood vessel growth	[16]
Chitosan derivatives nanoparticles	Methylcellulose disc	Reduction in number of blood vessels in contact with on-plants observed	[94]
Anti-VEGF Antibody	Methylcellulose disc	Visible anti-angiogenic activity observed through semi-quantitative evaluation	[169]
Anti-laminin antibody	Methylcellulose disc	Macroscopic observations saw a delay in capillary network development	[171]
Anginex	Plastic ring	Significant decrease in intersections of blood vessels with concentric rings projected onto images	[176,177]
Angiotensinogen	Plastic ring	First and second order centripetal blood vessels around on-plants were counted, with inhibition of smaller blood vessels observed	[178]
		Following FITC injection, blood vessel density, length and number of branch points were quantified highlighting inhibition of smaller blood vessels	[29]
Obtustatin (α1β1 inhibitor)		Decrease in the number of small new vessels growing towards on-plants	[179]

**Table 4:** Examples of cellular treatments/ tumours applied to CAM which affected angiogenesis.

Response	Treatment	Delivery Method	Angiogenic Outcome	Ref.
Pro-angiogenic	Glioblastoma cancer stem cells	Alginate scaffold	Increased blood vessel number converging towards on-plants	[114]
	Human umbilical vein endothelial cells (HUVECs)	Cylindrical scaffold	Increased number of blood vessels and blood vessel penetration into on-plant	[115]
	Adipose derived stem cells	Cylindrical scaffold	Increased number of blood vessels and blood vessel penetration into on-plant	[115]
		Hydrogel	Significant increase in vessel number, vessel length and vasculogenic index	[74]
		Seeded on a scaffold	Increased number of blood vessels converging towards on-plants	[108]
		Matrigel	Following von Willebrand factor staining and semi quantitative scoring, a significant increase in angiogenesis	[184]
	Burkitt’s Lymphoma cell lines (BL2B95 and BL74)	Matrigel	Following tissue sectioning increase in blood vessel diameter determined	[183]
	Human Liver Cancer (HepG2) cells	Matrigel	Increased number of blood vessels converging towards on-plants	[86]
	Prostate Cancer Cells (LNCaP)	Matrigel	A change in blood vessel number within a defined area observed	[185]
	Colon carcinoma (SW620)	Matrigel	Increase in angiogenic index was observed	[54]
	Neuroblastoma (NB15/FOXO3 cells)	Matrigel	Following desmin staining, increased micro-vessel formation was observed	[56]
	Glioblastoma (U87 MG) Cell lines	Matrigel	Increased observation of spoke wheel pattern of blood vessels radiating from on-plants	[55]
	Human Cardiopoietic Stem Cells	Scaffold	Blood vessel density within a defined area was increased	[186]
	Multiple myeloma plasma cells	Gelatin sponge	Induction of an increased vasculogenic index was calculated	[66]
	Mouse Melanoma (B-16)	Plastic ring	Development of visible spoke wheel pattern of blood vessels converging towards on-plants	[187]
	Human Melanoma (C8161)	Plastic ring	Significant increase in area occupied by endothelial cells observed within a defined area	[75]
		Hydrogel		
	Human Prostate Cancer (PC3)			
		Plastic ring		
	Skin graft	Plastic ring	Photobiomodulation along with cell application resulted in increased number of vascular junctions within a defined area	[104]
	Human Ovarian Tissue	Plastic ring	Visual estimation of area occupied by blood vessels compared to total surface area resulted increased angiogenesis and neovascularisation	[188]
	Melanoma Tumour Tissue	Tumour	Spoke wheel pattern of capillaries converging towards on-plants observed	[189]
	Recurrent respiratory papilloma tissue (RRP)	Tumour	Increase in blood vessel number within a defined area observed	[125]
	Hepatocellular Carcinoma Tumour tissue	Tumour	Increased micro vessel density within a defined area observed	[190]
	Human Malignant Ovarian tumours	Tumour	Increase in the pattern, density, and size of the CAM blood vessels near the tumour implants visible	[191]
	Adenocarcinoma Tumour Tissue	Tumour	Increase in the pattern, density, and size of the CAM blood vessels near the tumour implants visible	[191]
	Glioma cells (C6)	Injected	Macroscopic observations indicated tumours became vascularised by CAM blood vessels	[78]
	Pancreatic carcinoma (10AS)	Injectí	Macroscopic observations indicated tumours became vascularised by CAM blood vessels	[78]
Anti-angiogenic	Colon carcinoma (SW480)	Collagen	No Induction of angiogenesis or increased angiogenic index	[54]
	Burkitt’s Lymphoma cell lines (BL2)	Matrigel	Following tissue sectioning reduced blood vessel diameter observed	[183]

**Table 5:** Examples of treated and gene modified cells or conditioned media (CM) applied to CAM assay.

	Gene Modification	Cell Type	Delivery Method	Angiogenic Response	Ref.
Pro-angiogenic	FGF-1 expression plasmid	Bovine Endothelial Cells	Gelatin sponge	Following tissue sectioning, and staining for von Willebrand Factor, a twofold increase in capillary number was quantified	[135]
	LV miR-205 inhibition	Endothelial colony-forming cell CM	Not mentioned	Software quantified significant increase in blood vessel density within a defined area	[192]
	Sphingosine-1-phosphate treated	Osteoblast cell (MG-63) CM		Increase in blood vessel number within a defined area was quantified	[193]
	miR-338-3p inhibition plasmid	Hepatocellular carcinoma (HCC) CM	Filter disc	Visual inspection of second and third order blood vessels inferred increased blood vessel formation	[194]
	AGO2 expression plasmid	Myeloma cell CM		Significant increase in on-plant infiltrating blood vessels observed	[195]
	IFN-γ treated	Mesenchymal stem cell CM	Pipetted	A significant increase in number of small blood vessels (diameter less than 1 mm)	[196]
	TNF-α treated				
	IFN-γ and TNF-α treated			A significant increase in both small and large blood vessels	
Anti-angiogenic	AAV-Timp1- transduced	Chinese hamster ovary cells	Gelatin sponge	No spoke wheel pattern of blood vessels radiating from on-plants	[197]
	LV mediated Angiopoietin-2 shRNA	Pancreatic carcinoma cells	Pipetted	Decrease in number of blood vessel branch points	[198]
	Connective tissue growth factor (CTGF)-shRNA	OASF cell CM	Pipetted	Significant reduction in blood vessel count	[199]
	Endostatin expression plasmid	COS-1 cell CM	Pipetted	Significant reduction in blood vessel branch points	[200]
	LV VEGF shRNA	Hypertriploid renal cell carcinoma CM	Pipetted	Significant decrease in blood vessel counts and total blood vessel length	[201]
	CCL5-shRNA	Chondrosarcoma cells (JJ012)	Matrigel	Significant decrease in blood vessel branches	[202]
	Sema3C transfected	Glioblastoma cell line (U87 MG)	Collagen	Diminished observation of a spoke wheel pattern of blood vessels radiating from on-plants	[55]
	AGO2-shRNA	Myeloma cell CM	Filter disc	Lower blood vessel densities infiltrating the on-plants observed	[195]
	Vascular endothelial cell growth inhibitor (VEGI) expression plasmid	HeLa cell CM	Filter disc	Significant inhibition of neovascularization	[203]
	Novel immunotoxin (VEGF165-PE38) expression plasmid	HEK293 cell CM	Not mentioned	Inhibition in growth of capillary-like structures	[204]
	LV miR-205 OE	Endothelial colony-forming cell CM		Visual inspection saw reduced blood vessel formation	[192]
	miR-181a-5p expression plasmid	Fibrosarcoma (HT1080) cell CM	Gelatin sponge	Impairment of new blood vessel formation observed	[205]
	Nuclear Factor-Erythroid 2 (NRF2) shRNA	Human colon cancer cell CM	Matrigel	Significant reduction in blood vessel branch points in circular region around on-plants	[206]
	P53 Isoform (Δ133p53) deletion	Human Glioblastoma (U87) cell CM	Silicon ring	Following tissue sectioning and staining, reduced blood vessels quantified	[207]
	LV miR-542-5p	Non-small cell lung cancer CM	Silicon ring	Significant reduction in percentage vascular density	[109]

Key: CM: conditioned media; LV: Lentiviral; miR: microRNA; OE: Overexpression; shRNA: short hairpin RNA.

## Data Availability

Not applicable.

## Supplementary Materials

The following are available online at https://www.mdpi.com/article/10.3390/ijms23010452/s1.
